# High Dose Radiotherapy to Automated Implantable Cardioverter-Defibrillator: A Case Report and Review of the Literature

**DOI:** 10.1155/2014/989857

**Published:** 2014-09-07

**Authors:** Inaya Ahmed, Wei Zou, Salma K. Jabbour

**Affiliations:** Department of Radiation Oncology, Rutgers Cancer Institute of New Jersey, Rutgers University, New Brunswick, NJ 08903, USA

## Abstract

We report a case of successful full-dose chemoradiotherapy to stage IIIB nonsmall cell lung cancer (NSCLC) in a 59-year-old man with extensive cardiac history and an automated implantable cardioverter-defibrillator (AICD) located within the radiotherapeutic field. In this case, the AICD was a St. Jude Medical Fortify Assura VR 1257-40Q ICD, and it was implanted prophylactically during bypass grafting. Although we do not recommend routine radiotherapy dose to exceed recommended current guidelines due to the potential risks to the patient, this is a situation where relocation of the device was not possible. Fortunately, our patient was not AICD-dependent; so following much discussion and deliberation, the decision was made to treat the patient with AICD in place. The patient completed definitive chemoradiotherapy with concurrent cisplatin and etoposide and thoracic irradiation to 69.6 Gy. The minimum, maximum, and mean doses to the AICD directly were 13.5 Gy, 52.4 Gy, and 29.3 Gy, respectively. The device withstood full thoracic radiation dose, and the patient denied cardiac symptoms during the time before, during, and after completion of therapy. We sought to offer this case for both teaching and guidance in practice and to contribute to the published literature currently available in this area.

## 1. Introduction

The optimal protocol for providing radiotherapy to patients with pacemakers or AICDs remains controversial. Irradiation at or near the site of implanted cardiac devices may cause device malfunction, memory loss, deactivation, or resetting. The risks are most grave for device-dependent patients. However, radiation dose parameter recommendations have yet to be established for modern cardiac devices. Although the American Association of Physicists in Medicine (AAPM) published guidelines for irradiating patients with pacemakers as early as 1989, no guidelines pertaining to patients with AICDs existed until 2004 [1, 2]. The recommendations by Solan et al. were an amalgamation of existing guidelines from published literature, device manufacturers, and various institutions; and they were “intended to streamline the process of delivering RT to patients with ICPs (intracardiac pacemakers) and ICDs (intracardiac defibrillators) by proposing ‘universal precautions'” [2]. Another comprehensive set of guidelines was published by a Dutch group most recently in 2012 [3]. These guidelines vary slightly by risk group and offer dosing, monitoring, and safety recommendations for each risk group. We report a case of successful full-dose chemoradiotherapy to stage IIIB NSCLC in a man with extensive cardiac history and an AICD located within the radiotherapeutic field.

## 2. Case Report

A 59-year-old man, with extensive cardiac history and an AICD located within the radiotherapeutic field, was successfully treated for stage IIIB NSCLC with definitive dose of thoracic chemoradiation. With a history of type II diabetes mellitus and transient ischemic attacks, the patient was initially evaluated at our institution for cardiac disease. He was found to have triple vessel coronary artery disease and congestive heart failure requiring coronary artery bypass grafting (CABG) and placement of a prophylactic AICD (St. Jude Medical Fortify Assura VR 1257-40Q ICD). During preoperative assessment for CABG, an incidental left apical lung mass appeared on chest radiograph. The decision was made to follow the mass without intervention at that time.

Two months after surgery, he returned to the hospital for a fall during a hypoglycemic episode, at which point a chest radiograph revealed a persistent 3.0 by 2.2 cm mass in the left upper lobe as well as left hilar and supraclavicular lymphadenopathy, this time prompting evaluation for malignancy. Biopsy and staging studies confirmed T1bN3M0, stage IIIB poorly differentiated adenocarcinoma of the lung. Due to the complexities involved in radiation therapy (RT) to patients with AICDs, the final treatment plan included interdisciplinary input from radiation oncology, medical oncology, and cardiology.

**Figure 1:**
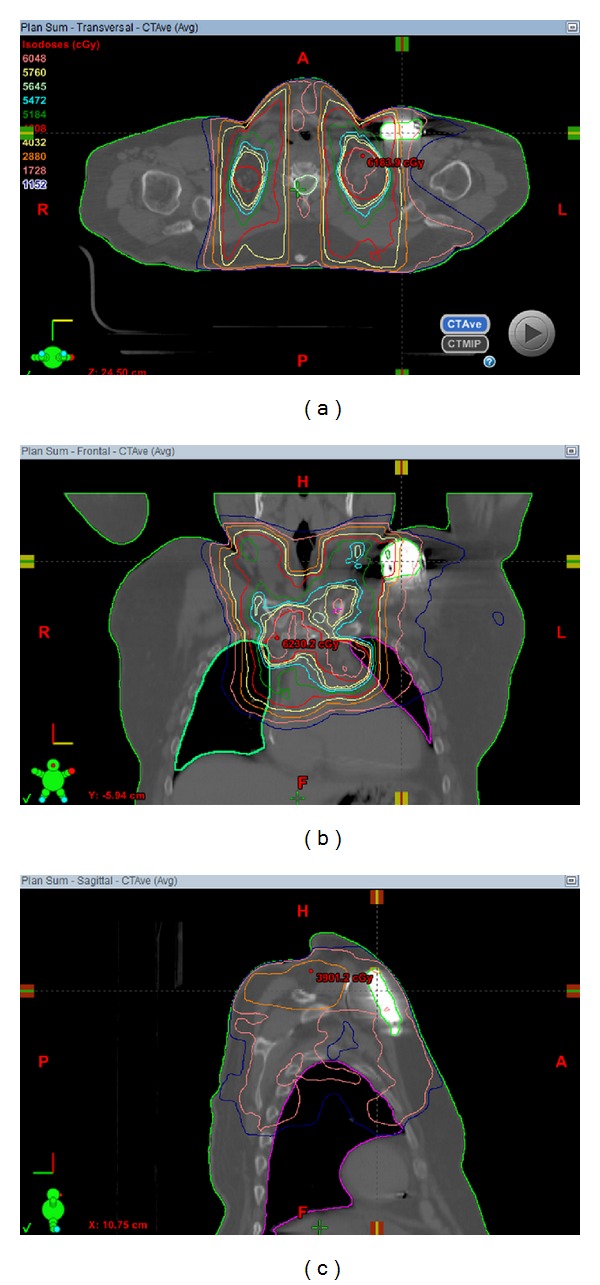
Axial (a), frontal (b), and sagittal (c) CT scans with overlying treatment volumes and corresponding doses outlined in color. AICD shown to be in field of RT.

The radiation oncology team hesitated to deliver RT to the patient with an AICD located within the field of radiation due to the risk of AICD malfunction. Multiple discussions were held with cardiology, St. Jude Medical, and with the physicians and radiation oncology physics teams about the patient. However, repositioning of the AICD was not possible in this patient's case. Fortunately, our patient was not AICD-dependent, as he had the device prophylactically implanted after CABG and had since experienced a single remote episode of ventricular tachycardia [4]. After thorough consultation and discussion among the physicians and physics team, the decision was made to treat the patient with AICD in place (Figures 1(a), 1(b), and 1(c)). AICD interrogations were obtained once before treatment, at least weekly during treatment, and once after treatment and the patient continues to be monitored by his cardiologist. During each fraction of RT, he was actively monitored by a nurse or medical doctor with blood pressure, heart rate, and pulse oximetry measurements while in the treatment room.

The patient completed definitive chemoradiotherapy with concurrent cisplatin and etoposide and thoracic irradiation to 69.6 Gy. He was first treated with 3-dimensional conformal technique to 30.6 Gy in 17 fractions using both 6 mV and 15 mV photons and an anteroposterior-posterioanterior combination, followed by intensity modulated RT to 27.0 Gy using 6 mV photons at 1.8 Gy per fraction for 15 fractions. He then received a boost of 12.0 Gy using 15 mV photons, delivered via stereotactic body RT in 4 fractions at 300 Gy per fraction. The minimum, maximum, and mean doses to the AICD directly were 13.5 Gy, 52.4 Gy, and 29.3 Gy, respectively.

He experienced no cardiac events before, during, or after treatment, although he remained tachycardic at 150 beats per minute during most of his treatment period, possibly related to dexamethasone during chemotherapy. The AICD maintained proper functionality, without resetting. The patient experienced expected toxicities of thoracic RT, all of which were grade 2 in severity: dysphagia, dermatitis, dyspnea, weight loss, and fatigue.

At his one month follow-up appointment, he reported feeling much better than he did during treatment, and he had gained back the 10 lbs he had lost during treatment. On review of systems, he reported no cardiac complaints since the end of treatment and his heart rate during the visit was down to 113 beats per minute. The AICD had maintained functionality throughout treatment. He is now 6 months out from treatment and the AICD shows no dysfunction.

## 3. Discussion

In the care of this patient, the physician team generally followed published recommendations before, during, and after treatment [2, 3]. However, we strayed from guidelines in ultimately keeping the AICD in place despite doses to the device >1.0 Gy, which was a deeply contemplated decision made by the patient's cardiologist and oncologists [2]. The Dutch guidelines categorized 0–2 Gy to the device as “low risk,” 2–10 Gy as “intermediate risk,” and >10 Gy as “high risk cases” for pacing-independent patients. For high risk patients, these guidelines propose relocation of implanted cardiac device or reconsideration of the necessity of radiotherapy [3]. Because AICDs are 5–10 times more sensitive than pacemakers to RT, irradiating the patient's AICD to a maximum of 52.4 Gy did indeed pose a risk of malfunction [5]. The fact that our patient was a poor surgical candidate who was fortunately not device-dependent—as it was implanted prophylactically—provided the greatest support for this treatment decision. Furthermore, no clear parameters have been established by manufacturers in terms of safe or acceptable doses to ICDs [6]. In addition, ionizing radiation has a cumulative effect on AICDs, which may manifest after completion of treatment. The patient's AICD reading after completion of treatment was negative for any abnormalities. Furthermore, he continues to see his cardiologist for continued interrogations of his AICD which remains without dysfunction 6 months later.

To our knowledge, this is the first reported case to be treated to full dose in the thoracic cavity with such a high dose directly to the AICD. In 2005, Hurkmans et al. published a study in the Netherlands examining dose tolerance in the latest generation of implantable cardioverter/defibrillator devices [7]. Eleven devices were directly irradiated by 6 mV photon beams, each to a cumulative dose of 20 Gy. Four of the 11 devices experienced complete loss of function after 1.5 Gy of radiation. To our knowledge, this is the highest dose to AICDs previously published in the literature, inside or outside the human body.

Gelblum and Amols investigated the effects of RT on AICDs in 33 patients [6]. The maximum dose that any patient received to the AICD was 3.0 Gy, and 1 patient experienced a complete reset of his AICD during RT to the pelvis after only 4 treatments with 15 mV photons. The AICD was not in the field of radiation. Once the patient was switched to 6 mV photons, no additional events occurred, and the patient received a total of 0.2 Gy to the AICD. The authors argue that the effect of radiation on AICDs is not dose driven, since all patients who received >2 Gy to their AICDs were free of device malfunction [6]. Yet, reports in the literature generally limit the radiotherapeutic dose to the AICD to ≤5.0 Gy (Makker, prospective Japanese, IMRT) [8–10].

We hope this report contributes to the available literature on outcomes of delivering RT to full dose in a patient with an AICD. Guidelines for treating these patients are not fully developed, and as implantable cardiac devices continue to modernize, additional study will be necessary for characterization of dose tolerance in these devices.

We do not recommend routine radiotherapy dose to exceed guidelines as stated above, due to the potential risks to the patient. Additionally, our patient had a newly implanted AICD, and effects may differ in devices that have remained in the body for longer periods of time. However, this is a situation where relocation of the device was not possible, and the device withstood full thoracic radiation dose. Such situations should be approached with extreme care and deliberation by the medical team. It should not be taken for granted that the AICD will remain unharmed in the setting of exceeding dose tolerances to the AICD. Had the patient been device-dependent and experienced dysfunction of his device, he could have experienced life threatening consequences or the device could have shorted or fired.

We sought to offer this case for both teaching and guidance in practice and to contribute to the published literature currently available in this area.
